# Immunomodulatory, Anticancer, and Antioxidative Activities of Bioactive Peptide Fractions from Enzymatically Hydrolyzed White Jellyfish (*Lobonema smithii*)

**DOI:** 10.3390/foods13213350

**Published:** 2024-10-22

**Authors:** Phitchapa Prommasith, Utoomporn Surayot, Narongchai Autsavapromporn, Weerawan Rod-in, Pornchai Rachtanapun, Sutee Wangtueai

**Affiliations:** 1 Faculty of Agro-Industry, Chiang Mai University, Chiang Mai 50100, Thailand; 2 Cluster of Innovation for Sustainable Seafood Industry and Value Chain Management, Chiang Mai University, Chiang Mai 50200, Thailand; 3 Division of Radiation Oncology, Department of Radiology, Faculty of Medicine, Chiang Mai University, Chiang Mai 50200, Thailand; 4 Department of Agricultural Science, Faculty of Agriculture Natural Resources and Environment, Naresuan University, Phitsanulok 65000, Thailand; 5 Division of Packaging Technology, School of Agro-Industry, Faculty of Agro-Industry, Chiang Mai University, Chiang Mai 50100, Thailand

**Keywords:** jellyfish, protein hydrolysate, peptides, antioxidant, immunomodulation, anticancer

## Abstract

This study aimed to develop bioactive protein hydrolysates from low-value edible jellyfish obtained from local fisheries using enzymatic hydrolysis. Fresh white jellyfish were hydrolyzed using several commercial proteases, including alcalase (WJH-Al), flavourzyme (WJH-Fl), and papain (WJH-Pa). The antioxidant, immunomodulatory, and anticancer activities of these white jellyfish hydrolysates (WJH) were investigated. The results demonstrated that the crude WJH exhibited strong antioxidant properties, including DPPH, ABTS, and hydroxyl radical scavenging activities, as well as ferric-reducing antioxidant power. Additionally, the hydrolysates showed notable immunomodulatory activity. However, all WJH samples displayed relatively low ability to inhibit HepG2 cell proliferation at the tested concentrations. Among the hydrolysates, WJH-Pa demonstrated the highest antioxidant and immunomodulatory activities and was therefore selected for further bioactive peptide isolation and characterization. Ultrafiltration membranes with three molecular weight (MW) cut-offs (1, 3, 10 kDa) were used for peptide fractionation from WJH-Pa. Six potential peptides were identified with the MW range of 1049–1292 Da, comprising 9–12 residues, which exhibited strong antioxidant and immunomodulatory activities.

## 1. Introduction

Edible jellyfish has long been used as a delicacy food in Asian countries, including Thailand, Japan, China, and South Korea [1]. In 2020, the total world production of captured edible jellyfish (*Rhopilema* spp. and *Stomolophus meleagris*) and aquaculture of edible red jellyfish (*Rhopilema esculentum*) was estimated to be ~250 and ~30 thousand tons of live weight/year, respectively [2]. Jellyfish processing into a human food source must happen shortly after they are harvested since jellyfish undergo rapid spoilage. Commercial jellyfish products are mostly brined and dried- or semi-dried-salted. Traditional processing is mostly used for jellyfish processing, which is done by using a mixture of salt and aluminum salt, as a preservative agent tastes acidic and its texture is crunchy and drier [3,4]. In Thailand, edible jellyfish are primarily captured by local fisheries in the Gulf of Thailand and the Andaman Sea, with one of the main species caught being the white jellyfish (*Lobonema smithii*). The captured jellyfish are sold to local processing plants located near the fisheries area, typically at a lower price ranging from approximately 0.5 to 0.7 USD/kg, depending on size. The primary jellyfish product for both in-country consumption and exportation is dried-salted jellyfish, generating an annual value of ~10 million USD [5]. Jellyfish have potential for human use beyond serving as a food resource. Efforts have been made to increase their value by leveraging their nutritional benefits. This advantage has garnered increased attention due to jellyfish containing collagen, protein, amino acid polysaccharides, and fatty acids, presenting new challenges and strategies for sustainable development in their utilization [5,6,7].

Bioactive protein hydrolysates are obtained from hydrolyzation of native protein using several methods such as chemical, physical, or enzyme processes. The bioactive proteins, which are primarily constituted of peptides with 2–20 amino acids, are being most commonly produced by enzymatic hydrolysis [8]. Presently, many research studies have reported on bioactive protein hydrolysates derived from native proteins of various marine invertebrates. Some reports have explored several bioactive properties of protein and collagen hydrolysates from jellyfish, such as jellyfish protein hydrolysate with antioxidant properties from *Rhizostoma pulmo*, salted *Nemopilema nomurai*, and *L. smithii* [5,9,10]; anti-tyrosinase activity from salted *L. smithii* [5]; anti-angiotensin-I-converting enzyme (ACE) activity from *R. esculentum* [11,12]; jellyfish collagen hydrolysate with antioxidant activity from *R. esculentum* and *R. hispidum* [13,14,15]; anti-tyrosinase activity from *R. hispidum* [15,16]; anti-fatigue activity from *R. esculentum* [14]; UV protection from *R. esculentum* [13]; immune effects from *N. nomurai* [17,18]; wound healing from *R. esculentum* [19]; and lipid-lowering activity from marinated *N. nomurai* [20]. Their biological activities depend on their production process and peptide characteristics, such as amino acid composition, peptide sequence, enzyme type, sample species, and appropriate hydrolysis conditions.

Therefore, the development of bioactive protein hydrolysates from edible jellyfish is potentially highly beneficial for sustainable marine food resource utilization. This study aimed to use low-value fresh jellyfish sourced from local fisheries to produce bioactive protein hydrolysates through enzymatic hydrolysis. The antioxidative, immunomodulatory, and anticancer activities of jellyfish protein hydrolysate were investigated and peptide isolation and characterization from the hydrolysate were carried out.

## 2. Materials and Methods

### 2.1. Fresh Jellyfish Preparation

The fresh *L. smithii*, edible white jellyfish, were purchased from a community fisherman in Palian District, Trang Province, Thailand. They were captured from the Andaman Sea area by coastal fisheries using manual traditional fishery equipment. After arrival at the fishing pier, jellyfish were kept in ice and shipped to the preparation area. The jellyfish were packed in polyethylene bags, frozen, placed in carton boxes (20–25 kg/box), and transported under a freezing temperature of −18 to −20 °C (within 12 h) to the Faculty of Agro-Industry, Chiang Mai University, located in Samut Sakhon Province, Thailand. After arriving, the jellyfish were kept in the freezing room until further use.

### 2.2. Enzymes and Chemicals

Flavourzyme (≥500 AU/g), Alcalase 2.4 L [≥2.4 AU/g], and Papain (≥3 activity units AU/mg), along with 1,1-diphenyl-1-picrylhydrazyl, 2,2’-azino-bis [3-ethylbenzothiazoline-6-sulfonic acid], 2,4,6-Tris(2-pyridyl)-s-triazine, ferrous sulfate heptahydrate, and ferrous sulfate heptahydrate were sourced from Sigma-Aldrich (St. Louis, MO, USA). The cell culture medium RPMI-1640 was sourced from Gibco (Thermo Fisher Scientific Inc., Waltham, MA, USA), while fetal bovine serum (FBS), penicillin, and streptomycin were purchased from Welgene (Daegu, Republic of Korea). All chemicals and reagents were analytical grade.

### 2.3. Determination of Raw Material Proximate Composition

Fresh jellyfish were analyzed to determine their proximate composition using the AOAC [21] method. The results of this method were 934.01, 954.01, 991.36, and 942.05 for moisture, crude protein, lipids, and ash contents, respectively. Briefly, moisture content was measured by drying a sample at 105 °C for 24 h in an oven (FD260, Binder, Tuttlingen, Germany). Crude protein content was measured using the Kjeldahl method. The nitrogen content and conversion factor (6.25) were calculated to obtain crude protein content. The Soxtex extraction method was used to determine the total lipid content. Ash content was determined by incinerating a sample placed in a porcelain crucible in a furnace at 550 °C.

### 2.4. Preparation of White Jellyfish Hydrolysate

Frozen white jellyfish were thawed in a refrigerator (4–5 °C), washed in running tap water to remove foreign matter, cut, re-washed, drained in a plastic basket, and chopped by using a kitchen blender (Blendforce BL438166, Tefal, Bangkok, Thailand). The optimum hydrolysis conditions for each enzyme (alcalase, flavourzyme, and papain) were determined based on our preliminary study using response surface methodology (RSM) with a face-centered composite design, which included two factors: enzyme concentration and hydrolysis time. These conditions were analyzed using multiple response optimization, considering yield, degree of hydrolysis (DH), and antioxidant activities (DPPH, ABTS, and H_2_O_2_ scavenging activities). The desirability function of the Design-Expert statistical program version 11 (Stat-Ease, Inc., Minneapolis, MN, USA) was applied to optimize hydrolysis conditions, with the maximum goal setting for all responses (DH, yield, DPPH, ABTS, and H_2_O_2_ scavenging activities). The results indicated that the optimal conditions for producing jellyfish hydrolysates involve enzymatic digestion with 5.0% papain for 343 min, 1.0% alcalase for 360 min, or 4.64% flavourzyme for 360 min. In brief, minced jellyfish was mixed with distilled water with a ratio of 1:2 (*w*/*v*), heated in a water bath at boiling temperature for 15 min to inhibit the endogenous enzyme and cooled to the optimum temperature for each enzyme. Alcalase, flavourzyme, and papain were individually added with each concentration (% *w*/*w* protein content) as above mentioned. Hydrolysis at the optimal time for each enzyme was conducted in a Memmert WNB45 shaking water bath (Schwabach, Germany) at the optimal temperatures: 60 °C for alcalase, and 50 °C for both flavourzyme and papain. Following hydrolysis, the reaction was halted by heating the mixture in boiling water for 15 min. Then, the mixture was cooled to room temperature using tap water and centrifuged at 5500× *g* for 15 min. The supernatant was collected and freeze-dried (GFD-3H freeze-dryer, Grisrianthong Co., Ltd., Samut Sakhon, Thailand). The freeze-dried hydrolysate was then packed in a zip-lock plastic bag and stored in a freezer (−20 °C) until further analysis.

### 2.5. Determination of Yield

The yield of jellyfish hydrolysate was calculated gravimetrically after freeze-drying using the following Equation (1).

(1) $$Yield(\%)=\left[\frac{freeze-dried\;jellyfish\;hydrolysate\;weight(g)}{fresh\;jellyfish\;weight(g)}\right]\times 100$$

### 2.6. Degree of Hydrolysis (DH)

The percentage of DH was measured using the protocol described by Doungapai et al. [22]. After fresh jellyfish were hydrolyzed into protein hydrolysate, 125 µL of solution was mixed with 2 mL of 0.2125 mol/L phosphate buffer (pH 8.2) and 1 mL of 0.01% TNBS (prepared with 0.2125 mol/L phosphate buffer, pH 8.2). The reaction was initiated by placing the mixture at 50 °C in the dark, followed by incubation for 30 min. To terminate the reaction, 2 mL of 0.1 mol/L sodium sulphite was added into the mixture and cooled at room temperature. A VarioskanTM LUX microplate reader (Thermo ScientificTM, Waltham, MA, USA) was then used to determine the absorbance at 420 nm. The percentage of DH was expressed using the following Equation (2).

(2) $$DH(\%)=\left[\frac{\left(L-L_{0}\right)}{\left(L_{max}-L_{0}\right)}\right]\times 100$$

where L is the amount of Leu equivalence in the jellyfish protein hydrolysate, L_0_ is the amount of Leu equivalence in the minced jellyfish, and L_max_ is the total amount of Leu equivalence in the minced jellyfish obtained after hydrolysis by 6 mol/L HCl at 100 °C for 24 h.

### 2.7. Determination of Antioxidative Activity

#### 2.7.1. DPPH Radical Scavenging Activity

DPPH radical scavenging activity was determined with the method described by Upata et al. [5]. The 0.1 mol/L DPPH solution was prepared by dissolving in 70% ethanol. The reaction mixture DPPH solution was added into the protein hydrolysate solution with a radio of 1:1 (*v*/*v*), kept in the dark for 30 min, and the absorbance at 517 nm was measured using microplate reader. DPPH radical scavenging activity was expressed using Equation (3), with the IC_50_ value (estimated from the linear regression graph) representing the concentration of the hydrolysate required to scavenge 50% of the DPPH radicals from the initial concentration.

(3) $$DPPH\;radical\;scavenging\;activity\;(\%)=\left[\frac{\left(Absorbance\;of\;control-Absorbance\;of\;sample\right)}{Absorbance\;of\;control}\right]\times 100$$

#### 2.7.2. ABTS Radical Scavenging Activity

ABTS radical scavenging activity was assessed using the method described by Upata et al. [5]. An ABTS solution was prepared by combining a 7 mmol/L ABTS solution with a 2.45 mmol/L potassium persulfate solution in a 1:1 (*v*/*v*) ratio, and then stored in the dark at 4 °C for 16–18 h. Prior to use, the ABTS solution was diluted with 70% ethanol to reach an absorbance of 0.7 ± 0.05 at 734 nm. Subsequently, 1900 µL of the ABTS solution was mixed with 100 µL of the protein hydrolysate solution and incubated in the dark for 8 min before obtaining absorbance at 734 nm. The ABTS radical scavenging activity was expressed using Equation (4) and reported as the IC_50_ value, similar to the DPPH analysis.

(4) $$ABTS\;radical\;scavenging\;capacity\;(\%)=\left[\frac{\left(Absorbance\;of\;control-Absorbance\;of\;sample\right)}{Absorbance\;of\;control}\right]\times 100$$

#### 2.7.3. Ferric Reducing Antioxidant Power (FRAP)

The FRAP assay was conducted according to the protocol described by Mongkonkamthorn et al. [23]. The FRAP solution was prepared by mixing 20 mmol/L FeCl_3_ in deionized water, 10 mmol/L TPTZ in 40 mmol/L HCl, and 300 mmol/L acetate buffer at a ratio of 1:1:10, respectively, followed by incubation at 37 °C in a water bath for 30 min. Then, 2850 µL of the FRAP solution was combined with 150 µL of the protein hydrolysate solution and allowed to react for 30 min. The absorbance was measured at 593 nm. A standard curve using solutions of 0–10 mmol FeSO_4_·7H_2_O was prepared to express the FRAP value as mmol FeSO_4_ per gram of sample. Additional dilutions were done if the FRAP value was over the linear range of the standard curve.

#### 2.7.4. Hydroxyl Radical (OH) Scavenging Activity

The OH radical scavenging activity was conducted according to the method described by Guo et al. [24]. The 2 mL of protein hydrolysate solution was mixed with 1 mL 1,10-phenanthroline solution (1.865 mmol/L, dissolved in 0.1 mol/L phosphate buffer, pH 7.4) and 1 mL 1.865 mmol/L FeSO_4_·7H_2_O solution. Then, the reaction was activated by adding 1 mL 0.03% (*v*/*v*) H_2_O_2_ and incubated in a water bath at 37 °C for 60 min. The absorbance of the mixture was measured at 536 nm. The OH radical scavenging activity was calculated using Equation (5). Distilled water and H_2_O_2_ were used to replace the sample for negative control and blank, respectively. The OH radical scavenging activity was represented in the IC_50_ value, similar to the DPPH analysis.

(5) $$OH\;radical\;scavenging\;activity\;(\%)=\left[\frac{\left(Absorbance\;of\;sample-Absorbance\;of\;negative\;control\right)}{\left(Absorbance\;of\;blank-Absorbance\;of\;negative\;control\right)}\right]\times 100$$

### 2.8. Determination of Anticancer Activity

The anticancer activity of jellyfish hydrolysate was evaluated by assessing its effect on inhibiting the proliferation of the HepG2 liver cancer cell line, following the modified method of Saiwong et al. [25]. The activity was reported based on HepG2 cell viability.

#### 2.8.1. HepG2 Cell Culture and Testing

The HepG2 cell line (Korean Cell Line Bank, Seoul, Republic of Korea) was seeded in RPMI-1640 medium with 10% FBS and 1% penicillin-streptomycin. The cells were incubated in an incubator with high humidity and 5% CO_2_ at 37 °C. They were then treated with either crude or fractionated jellyfish hydrolysate for 24 h. The treated sample concentrations of 1000, 2000, 3000, 4000, 5000, and 6000 µg/mL were tested, while 5-Fluorouracil (25 µg/mL) was used for a positive control.

#### 2.8.2. HepG2 Cell Viability Analysis

The WST assay was used to measure the proliferation rate of HepG2 cells (EZ-Cytox Cell Viability Assay Kit, DaeilLab Service, Seoul, Republic of Korea). Cell lines were placed in 96-well plates at 1 × 10^6^ cells/well (100 µL/well) and incubated with various concentrations of crude or fractioned jellyfish hydrolysate. The quantity of tetrazolium salt was measured at 450 nm using a microplate reader (EL-800, BioTek Instruments, Winooski, VT, USA).

### 2.9. Evaluation of Immunoregulatory Activity

#### 2.9.1. Culturing and Treating Macrophage Cell Lines

The RAW264.7 macrophage cell lines (Korean Cell Line Bank) were plated in RPMI-1640 medium supplemented with 10% FBS and 1% penicillin-streptomycin and were kept in a humidified incubator with 5% CO_2_ at 37 °C. RAW264.7. Cells were treated with 50, 100, 250, 500, 750, and 1000 µg/mL of crude or fractioned jellyfish hydrolysate for 24 h.

#### 2.9.2. Determination of RAW264.7 Cell Viability

The WST assay was applied to measure the proliferation rate of RAW264.7 cells in a similar manner to HepG2 cell viability.

#### 2.9.3. Determination of NO Production

The concentration of NO was determined using the Griess reagent (Sigma-Aldrich, St. Louis, MO, USA). RAW 264.7 macrophage cells were plated in 96-well plates at 1 × 10^6^ cells/well (100 µL/well) and incubated for 24 h. The cells were then incubated with samples at concentrations of 50, 100, 250, 500, 750, and 1000 µg/mL and incubated for an additional 24 h. Following treatment, the supernatant was mixed with 100 µL of Griess reagent and incubated for 10 min. The quantity of NO production was obtained by measuring the absorbance at 540 nm using the EL-800 microplate reader (BioTek Instruments, Winooski, VT, USA) and a reference curve of sodium nitrite.

### 2.10. Fractionation of Jellyfish Hydrolysate

The fractionation of crude jellyfish hydrolysate obtained from hydrolysis with three different enzymes was carried out according to a slightly modified method of Doungapai et al. [22]. The freeze-dried hydrolysates were dissolved in distilled water to obtain a solution of 5% (*w*/*v*) concentration, loaded into the 200 mL stirred cell (Amicon^®^, Merck KGaA), and fractionated using a series of nominal ultrafiltration membranes (Merck KGaA, Darmstadt, Germany) with MW cut-offs of 1, 3, and 10 kDa.

### 2.11. Peptide Isolation and Identification

Peptide isolation and identification was done following the method of Doungapai et al. [22] and Krobthong and Yingchutrakul [26]. The selected peptide fraction from Section 2.10 was lyophilized to obtain the powder. The lyophilized peptide fraction was dissolved in formic acid (0.1%) in water to achieve a concentration of 0.1 µg/µL and analyzed using an Orbitrap HF mass spectrometer (Thermo Scientific, MA, USA) with an ESI ion source set at 3.2 kV. A 5 µL sample of peptide solution was injected onto a C18 column (Thermo Scientific), with the column temperature kept at 60 °C throughout the separation process. Mobile phase was the gradient using 0.1% formic acid in water (mobile phase A) and 0.1% formic acid in 80% acetonitrile (mobile phase B), with a Linear gradient of 5–60% B in 60 min at 250 nL/min flow rate. Each analysis lasted 120 min. MS spectral data were collected using a Top10 method, which dynamically selects the most abundant precursor ions from a broad survey scan range (100–1400 *m*/*z*) with charge states ranging from +1 to +5. Precursor ions were isolated with a 1.4 *m*/*z* window, and MS/MS scans were performed with a starting mass of 120 *m*/*z*. The resolution for fragmentation spectra was set to 30,000 at *m*/*z* 200. The de novo sequencing algorithms with PeakX Studio 10.0 software (Bioinformatics Solutions Inc., Waterloo, ON, Canada) were used to identify the peptide mass and amino acid sequence from the MS/MS spectrum (two replicate LC-MS analyses). The 20-ppm peptide mass tolerance and the MS/MS tolerance was set to 0.2 Da. The 1% false discovery rate was used in the peptide filtering process for achieving high-confidence peptide identification. The sequences with highest abundance were reported. The acceptability of de novo peptide sequences was determined by applying a filter for an average local confidence (ALC) of ≥90%.

### 2.12. Statistical Analysis

All data are presented as the mean ± standard deviation (SD) based on triplicate measurements (*n* = 3). The differences between data groups were analyzed using one-way analysis of variance (ANOVA) followed by Duncan’s multiple range test, performed with SPSS version 17 software (SPSS, Inc., Chicago, IL, USA). The *p*-value (≤0.05) was applied for statistical significance.

## 3. Results and Discussion

### 3.1. Proximate Composition of Fresh White Jellyfish

Fresh white jellyfish contained 96.56 ± 0.02% (wet basis; wb) moisture and ~5% dry matter. Organic and inorganic matter in fresh white jellyfish showed the content of ash, protein, carbohydrate, and lipid as 65.14 ± 3.49, 21.52 ± 0.84, 9.91 ± 0.84, and 0.11 ± 0.01% of dry basis (db), respectively. The present results are consistent with the findings reported by Doyle et al. [27], which showed that the proximate composition of *C. capillata* was 95.8 ± 0.2% moisture (wet basis) and 76.8 ± 2.0% (db) ash, 16.5 ± 3.05% (db) protein, 0.88 ± 0.02% (db) carbohydrate, and 0.5 ± 0.1% (db) lipid content. Raposo et al. [4] reported that >95–98% of the wet weight matter in fresh jellyfish mainly contained water, while the dried weight matter comprised ash content, especially the jellyfish that live in brackish and marine waters which have a higher mineral concentration. In general, the protein content in jellyfish is over 50% collagenous protein, with ~33% of total amino acids being essential amino acids, and ~21% being non-essential amino acids [14,28].

### 3.2. White Jellyfish Hydrolysate (WJH) and Antioxidant Properties

The yields of WJH-Al, WJH-Fl, and WJH-Pa are presented in Table 1. No significant differences were observed among the yields obtained with the three enzymes, which ranged from approximately 2.2–2.5% (wb). This was a slightly lower yield than a previous study by Upata et al. [5], in which they used a similar enzyme but different hydrolysis conditions to produce a hydrolysate from the salted jellyfish. The present study obtained a lower yield, which might be affected by the difference of raw material used and the hydrolysis conditions. Types and concentration of enzyme and hydrolysis times played crucial roles in the enzymatic reaction in the protein hydrolysis process, as well as additional factors, such as the types and properties of the original protein and the optimum hydrolysis conditions [25,29].

The DH of WJH-Al, WJH-Fl, and WJH-Pa were significantly different (*p* ≤ 0.05) with a range of ~28–69% (Table 1). The peptide bonds in jellyfish protein structures were hydrolyzed during alcalase, flavourzyme, and papain hydrolysis, resulting in peptides and free amino acids. DH indicates the efficiency of enzyme activity in the protein hydrolysis process, measured by the breakdown of peptide bonds during enzymatic hydrolysis [8]. This study showed a slightly different DH for each enzyme. Flavourzyme is a blend of endo- and exopeptidases, which facilitates the production of free peptides and amino acids. In contrast, papain and alcalase are endopeptidases that specialize in hydrolyzing proteins by targeting peptide bonds, with a particular preference for uncharged residues [5,30].

The antioxidant activities of crude WJH-Al, WJH-Fl, and WJH-Pa are shown in Table 1. Antioxidant activities were determined with a FRAP value and the IC_50_ of DPPH, ABTS, and OH radical scavenging activities. The IC_50_ of DPPH radical scavenging activity was 0.45–4.61 mg/mL without a significant difference between WJH-Fl and WJH-Al (*p* > 0.05), which showed similar efficiency. DPPH, distinguished by its unpaired electrons, has the capacity to capture protons upon encountering antioxidants [31]. For the ABTS radical scavenging activity, three hydrolysates were in the range of ~2–5 mg/mL, of which the WJH-Al and WJH-Pa had the strongest activity, without significant difference (*p* > 0.05). Discrepancies between the IC_50_ values obtained from the ABTS and DPPH assays may occur because the ABTS assay is effective for evaluating both hydrophobic and hydrophilic antioxidants, whereas the DPPH analysis is primarily suited for hydrophilic antioxidants. Furthermore, variations in results might also arise from differences in the peptide and amino acid sequences of the antioxidant substances [32]. For the FRAP assay, the WJH-Al had the highest FRAP value at about 6.4 mmol FeSO_4_/g sample (*p* ≤ 0.05), while WJH-Fl and WJH-Pa were not significantly different (*p* > 0.05). The FRAP analysis evaluates antioxidant capacity by measuring the reduction of Fe^3+^ to Fe^2+^ under acidic conditions, comparing the total reducing power of the antioxidant compound to that of FeSO_4_ standard [33]. For OH radical scavenging activity, the strongest was observed in WJH-Fl (~2.7 mg/mL), with no significant difference in IC_50_ value between WJH-Al and WJH-Pa (*p* > 0.05). In the reaction system, H_2_O_2_ reacts with Fe^2+^ to release OH radicals. These radicals then convert Fe^2+^ to Fe^3+^, and only Fe^2+^ can react with 1,10-phenanthroline to produce a red compound (maximum absorbance at 536 nm). The level of decolorization in the reaction solution indicates the concentration of OH radicals [34].

### 3.3. Anticancer Activity of Crude WJH

**Figure 1:**
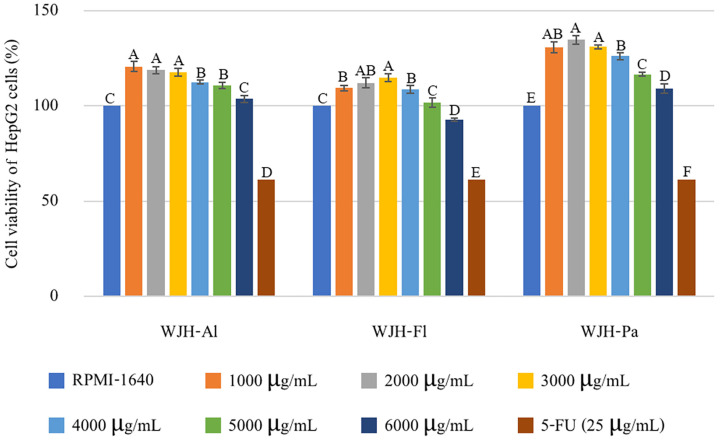
The cell viability of HepG2 cells treated with WJH-Al, WJH-Fl, and WJH-Pa; bars represent standard deviation, with triplicate (*n* = 3). Different upper letters on the bars indicate significant difference between groups (*p* ≤ 0.05).

The effects of crude WJH on cytotoxicity to the HepG2 liver cancer cell line was determined. Various concentrations of WJH-Al, WJH-Fl, and WJH-Pa (0–6000 µg/mL) and positive control (25 µg/mL of 5-Fluorouracil) were applied to HepG2 cells, with results shown in Figure 1. An increase of HepG2 cell viability was observed at increasing WJH concentrations (1000–4000 µg/mL), and cell viability subsequently decreased significantly (*p* ≤ 0.05), but the cell viability was higher than the control that went without treatment. This finding aligns with the previous study of Khalil et al. [35], wherein jellyfish extracts (25–200 µg/mL) demonstrated an increase in cell viability for human neuroblastoma and L929 fibroblast cell lines. Sea cucumber protein hydrolysate inhibited HepG2 cell proliferation by 40% when treated at a concentration of 500 μg/mL [25]. In these results, only the WJH-Fl (6000 µg/mL) showed decreased HepG2 cell viability to 90%, while the 5-fluorouracil showed about 60% cell viability. This suggests that WJH displayed relatively low ability to inhibit HepG2 cell proliferation at the tested concentrations. The concentration of the protein hydrolysate has been reported in improving the effectiveness of cytotoxicity in tumor cells by increasing the overall positive charge. Ion charges were one of the reasons that caused protein hydrolysate to bind with phospholipids on the cell membrane, resulting in cancer cell death [36,37]. Some protein hydrolysates can encourage cancer cell proliferation on the cell membrane, while others can prevent cancer cell development within the cell. The mechanism of action of peptides with opposite effects both inside and outside the cell depends on the type and characteristics of the cell [38].

### 3.4. Effects of Crude WJH on Macrophage Viability and Immunomodulatory Activity

**Figure 2:**
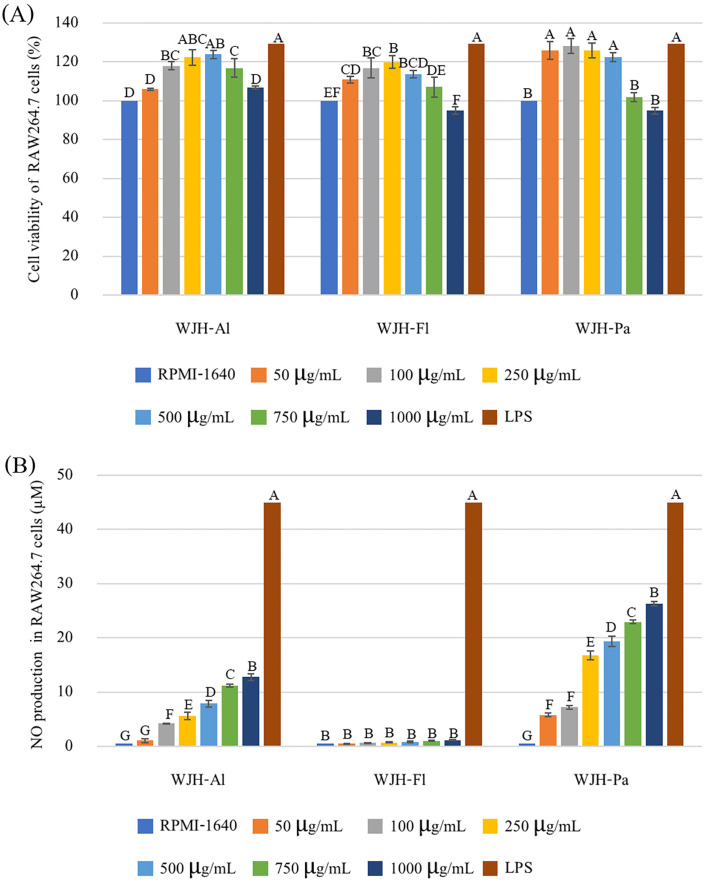
Effects of crude WJH on RAW264.7 cell viability (**A**) and NO production (**B**); bars represent standard deviation, with triplicate (*n* = 3). Different upper letters on the bars indicate significant difference between groups (*p* ≤ 0.05).

Macrophages serve as vital immunomodulatory cells, playing a crucial role in maintaining immune system balance and offering defense against invading pathogens [39]. A WST assay showed that WJH-Al, WJH-Fl, and WJH-Pa were similarly not cytotoxic in RAW264.7 cells at test concentrations ranging from 50 to 1000 µg/mL compared to the negative control (without treatment) and enhanced cell proliferation (Figure 2A). WJH-Al at concentrations of 100–750 µg/mL, WJH-Fl at concentrations of 50–500 µg/mL, and WJH-Pa at concentrations of 50–500 µg/mL showed a significant capability to enhance cell growth (*p* ≤ 0.05). Additionally, the effect of WJH on immunomodulatory activity was determined on RAW264.7 cells. The RAW264.7 cells treated with WJH-Al, WJH-Fl, and WJH-Pa (50–1000 μg/mL) showed varying levels of NO production, as illustrated in Figure 2B. WJH-Al and WJH-Pa demonstrated an increase in NO release with increasing concentration of treatment, with the highest NO production observed at a treatment concentration of 1000 µg/mL, while WJH-Fl showed the lowest NO release. Both WJH-Al and WJH-Pa induced highest NO release at ~12 and ~25 μM, respectively. The WJH-Pa induced the RAW264.7 cells to produce NO higher than WJH-Al by 2.06 times. Therefore, considering the overall bioactivities of WJH, WJH-Pa was selected for further study for fractionation and peptide characteristics.

### 3.5. Fractionation of WJH

WJH-Pa was selected for fractionation based on exhibiting the highest ABTS radical scavenging activity and immunomodulatory activity. An ultrafiltration membrane with varying molecular weight cut-offs was used to obtain the following fractions: WJH-Pa-I (>10 kDa), WJH-Pa-II (3–10 kDa), WJH-Pa-III (1–3 kDa), and WJH-Pa-IV (<1 kDa). Also, Sae-Leaw et al. [40] chose the highest ABTS radical scavenging activity of crude hydrolysate from seabass skin to be further studied.

**Figure 3:**
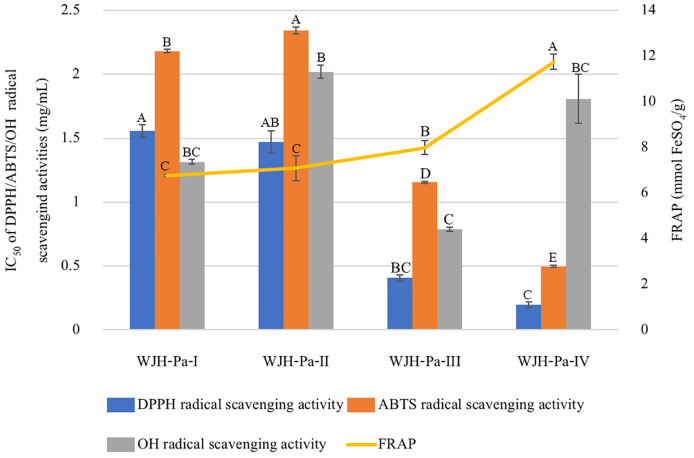
Antioxidant activities of crude and fractioned WJH-Pa; bars represent standard deviation, with triplicate (*n* = 3). Different upper letters on the bars and the line indicate significant difference between groups (*p* ≤ 0.05).

Figure 3 shows the antioxidative activities of WJH-Pa; all the fractions expressed free radical scavenging abilities. The WJH-Pa-IV fraction exhibited the best antioxidative activity of DPPH, ABTS radical scavenging activity, and FRAP, while the WJH-Pa-III fraction exhibited the best antioxidative activity of OH radical scavenging activity. The results indicated that the molecular mass of the protein hydrolysate was key to enhancing the antioxidant activity, in which protein hydrolysate with a small MW could exhibit higher bioactive and antioxidant activities than protein hydrolysate with larger MW. According to other studies, they reported that protein hydrolysate from blood clam [41], tuna dark meat [8], and jellyfish [11] with MWCO < 1 kDa exhibited higher antioxidant activities.

### 3.6. Effects of Fractioned WJH on Macrophage Cell Viability and Immunomodulatory Activity

**Figure 4:**
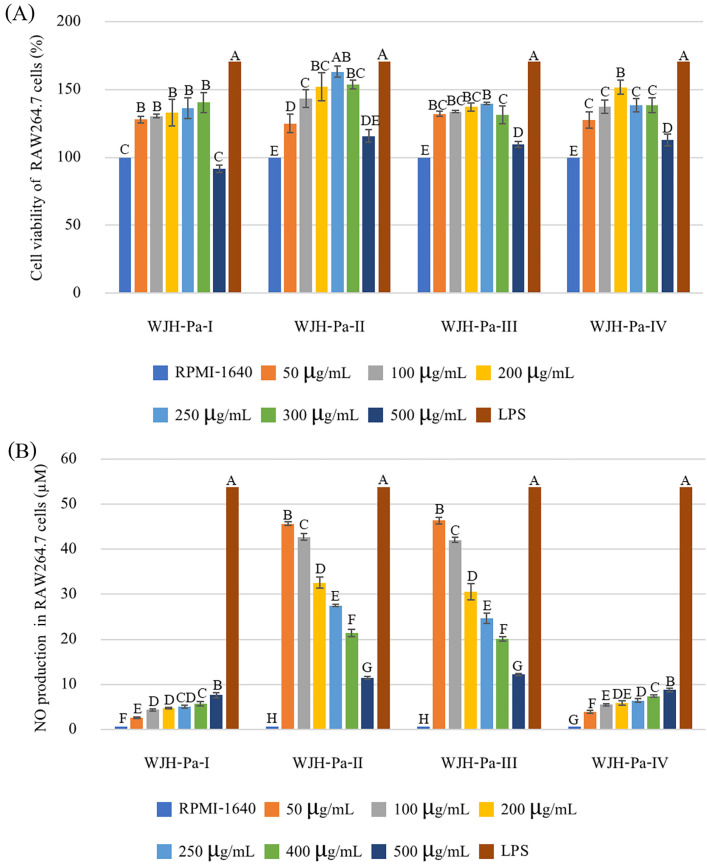
Effects of fractioned WJH-Pa on RAW264.7 cell viability (**A**) and NO production (**B**); bars represent standard deviation, with triplicate (*n* = 3). Different upper letters on the bars indicate significant difference between groups (*p* ≤ 0.05).

Most of the WJH-Pa fractions had no toxic effect on RAW264.7 cells of all fractions at concentrations ranging from 50–500 µg/mL (Figure 4A), of which only 500 µg/mL of WJH-Pa-I showed a slight decreasing of cell viability to 90%. WJH-Pa-II, at a concentration of 250 µg/mL, exhibited the highest proliferation rate, which was 1.63 times of the negative control (without WJH-Pa). Moreover, WJH-Pa (at concentrations of 50–300 µg/mL) significantly promoted cell viability in a dose-dependent manner (*p* ≤ 0.05). In particular, the lower molecular weight fractions (WJH-Pa-II, WJH-Pa-III, and WJH-Pa-IV) increased cell viability more than the control (without WJH fractions). However, at concentrations higher than 300 µg/mL, a slight decrease in cell viability was observed. In comparison, the higher molecular weight fraction (WJH-Pa-I) and a higher concentration (500 µg/mL) reduced RAW264.7 cell viability to 90%. The present results were consistent with Li et al. [42] and Yu et al. [43], which reported the low MW peptides exhibiting a higher RAW264.7 cell proliferation. The amount of NO production in RAW264.7 cells treated with WJH-Pa fractions was significantly different among fractions and concentrations (*p* ≤ 0.05, Figure 4B). The WJH-Pa-I and WJH-Pa-IV fractions promoted higher NO production in RAW264.7 cells at higher applied peptide concentrations. WJH-Pa-III fractions induced RAW264.7 cells release the greatest amount of NO when stimulated by WJH-Pa-III at the lowest concentration (50 µg/mL) with 46.37 µM of NO. NO is an important component of cellular communication involved in vasodilation, blood pressure regulation, neurotransmission, and the host immune defense system. However, excessive NO can have pathological effects, such as hypotension, severe inflammation, and cell damage [44,45]. Then based on the bioactivities, antioxidant activities and immunomodulatory activity, the fraction of WJH-Pa-III was selected to study the peptide characteristics.

### 3.7. Peptide Characterization

WJH-Pa-III was subjected to peptide sequencing using LC-MS/MS. Based on the identified peptide sequences with a de novo peptide sequencing score and ALC of ≥90%, six peptides were obtained, as shown in Table 2. The six peptides with a potent antioxidant and immunomodulatory effect were NPTSVVDLTK (1072.6 Da), FDTPSDFVK (1054.5 Da), PGGVGGLARYT (1046.6 Da), EPVPERPVK (1049.6 Da), LADQSRNPHSAP (1291.6 Da), and LPEWFPELGL (1199.6 Da). Each peptide contained between 9 and 12 amino acid residues, with molecular weights ranging from 1049.0 to 1292.0 Da (average: 1119.1 ± 102.65 Da). Bioactive protein hydrolysates generally consist of 2 to 20 amino acids per sequence, derived from the parent protein [46]. Therefore, they can easily penetrate the intestinal barrier to present their biological activities [47]. Immunomodulatory activity from purified peptide was influenced by amino acid content, peptide sequence, length, charged ion, hydrophobicity, and their structure [44]. Previous studies have reported that immunomodulatory peptides predominantly contain hydrophobic amino acids such as tyrosine (Y), glycine (G), leucine (L), alanine (A), valine (V), and proline (P), which may enhance peptide interaction with cell membranes and improve immunomodulatory activity [48,49,50]. In this study, hydrophobic amino acids were present in the selected peptide sequences, making up more than 50% of the total amino acids. This may enhance immune response activity. The hydrophobic amino acids most prevalent in the selected peptides were proline (P), valine (V), and leucine (L), accounting for 29%, 17%, and 17% of the total amino acids in the sequences, respectively. Additionally, an abundance of basic or hydrophobic amino acids at the end terminals is also associated with immunomodulatory activity [51]. Hydrophobic amino acids act as hydrogen donors, enhancing antioxidant activity by neutralizing unpaired electrons or radicals [40,49]. Moreover, some peptides which contain tryptophan, tyrosine, lysine, and arginine residue in the *C*-terminus could act as antioxidants [52,53]. An aromatic amino acid, tyrosine, can pair with free radicals by donating protons during radical scavenging activity and it also exhibits immune effects [44,54]. Other immunomodulatory and antioxidation peptides have been identified such as GAGLPGKRER (1039.56 Da) from *Pinctada fucata* muscle [55], HIAEEADRK (1068.15 Da) and AEQAESDKK (1005.04 Da) from tuna trimming protein hydrolysate [56], and RVKGKILAKRLN from *Sipunculus nudus* L. protein hydrolysate [57].

## 4. Conclusions

Bioactive protein hydrolysates from fresh white jellyfish were successfully produced by different enzymes. White jellyfish hydrolysate obtained by papain hydrolysis (WJH-Pa) exhibited greater antioxidant and immunomodulatory activities. WJH-Pa was selected for bioactive peptide fractionation and characterization, and six potential peptides were identified with molecular weights ranging from 1049 to 1292 Da, comprising 9 to 12 amino acid residues. Therefore, the low-value edible jellyfish obtained from local fisheries can potentially be used to produce bioactive peptides through papain hydrolysis, which can be incorporated as functional ingredients in food and nutraceutical products. However, it is recommended that further studies explore the reaction mechanisms and bioavailability through animal or human trials. Additionally, similar experiments using synthetic peptides might be conducted to confirm their potential bioactivities.

## Figures and Tables

**Table 1:** Yield (%), DH (%), and antioxidant activities of WJHs with different enzymes.

Jellyfish Hydrolysate	Yield (%)	DH (%)	Antioxidant Activities			
			DPPH (IC_50_) (mg/mL)	ABTS (IC_50_) (mg/mL)	FRAP(mmol FeSO_4_/g)	OH (IC_50_) (mg/mL)
WJH-Al	2.20 ± 0.50 ^A^	28.15 ± 2.49 ^C^	1.97 ± 0.80 ^B^	1.98 ± 0.01 ^B^	6.35 ± 0.07 ^A^	10.20 ± 0.08 ^A^
WJH-Fl	2.53 ± 0.08 ^A^	44.97 ± 0.48 ^B^	0.45 ± 0.07 ^B^	4.98 ± 0.20 ^A^	5.37 ± 0.32 ^B^	2.74 ± 0.20 ^B^
WJH-Pa	2.37 ± 0.30 ^A^	68.55 ± 2.13 ^A^	4.61 ± 1.10 ^A^	2.04 ± 0.01 ^B^	5.10 ± 0.13 ^B^	9.94 ± 0.91 ^A^

Note: WJH-Al: jellyfish protein hydrolysate using alcalase, WJH-Fl: jellyfish protein hydrolysate using flavourzyme, WJH-Pa: jellyfish protein hydrolysate using papain; all the values are mean ± SD with triplicate. Different upper letters in the same column indicate significant difference between groups (*p* ≤ 0.05).

**Table 2:** The amino acid sequences in the selected peptide fraction (WJH-Pa-III) from white jellyfish hydrolysate.

Peptide Hydrolysate Sequences	Molecular Mass (Da)	Length	ALC (%)	Charged Ion (*m*/*z*)
NPTSVVDLTK	1072.6	10	97	537.2947
FDTPSDFVK	1054.5	9	92	528.2547
PGGVGGLARYT	1046.6	11	91	524.2874
EPVPERPVK	1049.6	9	91	525.7994
LADQSRNPHSAP	1291.6	12	90	646.8267
LPEWFPELGL	1199.6	10	90	400.8833

Note: *m*/*z*: experimental mass; ALC: average local confidence.

## Data Availability

The original contributions presented in the study are included in the article, further inquiries can be directed to the corresponding author.
